# Computer-Aided Designing and Manufacturing of Lingual Fixed Orthodontic Appliance Using 2D/3D Registration Software and Rapid Prototyping

**DOI:** 10.1155/2014/164164

**Published:** 2014-05-11

**Authors:** Soon-Yong Kwon, Yong Kim, Hyo-Won Ahn, Ki-Beom Kim, Kyu-Rhim Chung, Seong-Hun Kim (Sunny)

**Affiliations:** ^1^Department of Orthodontics, School of Dentistry, Kyung Hee University, No. 1 Hoegi-dong, Dongdaemun-gu, Seoul 130-701, Republic of Korea; ^2^Department of Orthodontics, Center for Advanced Dental Education, Saint Louis University, 3320 Rutger Street, Saint Louis, MO 63104, USA; ^3^Department of Orthodontics, School of Medicine, Ajou University, No. 1 Wonchun-dong, Yeongtong-gu, Suwon 443-380, Republic of Korea

## Abstract

The availability of 3D dental model scanning technology, combined with the ability to register CBCT data with digital models, has enabled the fabrication of orthognathic surgical CAD/CAM designed splints, customized brackets, and indirect bonding systems. In this study, custom lingual orthodontic appliances were virtually designed by merging 3D model images with lateral and posterior-anterior cephalograms. By exporting design information to 3D CAD software, we have produced a stereolithographic prototype and converted it into a cobalt-chrome alloy appliance as a way of combining traditional prosthetic investment and cast techniques. While the bonding procedure of the appliance could be reinforced, CAD technology simplified the fabrication process by eliminating the soldering phase. This report describes CAD/CAM fabrication of the complex anteroposterior lingual bonded retraction appliance for intrusive retraction of the maxillary anterior dentition. Furthermore, the CAD/CAM method eliminates the extra step of determining the lever arm on the lateral cephalograms and subsequent design modifications on the study model.

## 1. Introduction

Advances in digital imaging systems, computer-aided design, and computer-aided manufacturing (CAD/CAM) technology are providing new possibilities in orthodontics. The application of CAD/CAM for establishing a virtual setup and fabricating transfer tray/jigs [1–3] has greatly improved the indirect bonding process. CAD/CAM has also enabled 3D virtual diagnosis, treatment planning, wafer fabrication, and customized bracket design [4–7]. Its use in orthognathic surgery has shown multiple advantages including reducing laboratory time for making surgical splints and improving accuracy for repositioning of the maxilla and mandible. Although the lingual orthodontic appliance provides distinctive esthetic advantages, its use has been limited due to increased chair time and more difficult mechanical control. Application of lingual orthodontic appliances is becoming easier with new technologies such as virtual positioning of the brackets and indirect bonding systems which utilize virtual setup models.

Accurate surface imaging is required to digitally manufacture orthodontic appliances. Even when CBCT scans are used for the diagnosis or design of an appliance, separate surface imaging of the dentition is required to compensate for poor surface rendering in the CBCT. Surface images of the dentition are typically obtained from a 3D optical scanner and registered with a CBCT scan. However, taking a CBCT solely for the fabrication of an orthodontic appliance is impractical considering the expense and radiation dose. Recently, 3D dental CAD/CAM solution software utilizing 2D lateral and posteroanterior (PA) cephalograms and 3D virtual dental models (3Txer version 2.5, Orapix, Seoul, Korea) has been introduced. Choi et al. evaluated the accuracy of orthognathic surgical wafers fabricated using the software and concluded that the new method using the cephalograms and surface scan can be regarded as an effective alternative for conventional 3D surface scan and CBCT methods [7].

The lateral cephalogram is important in designing orthodontic appliances for en-masse retraction of the maxillary anterior dentition. The lever arm length of the appliance is determined by the location of the center of resistance of the maxillary anterior teeth on the lateral cephalogram. The appliance design is then drawn on the study model. However, there is room for error when transferring design information from the lateral cephalogram to the actual study model.

On the contrary, the CAD/CAM method can precisely transfer the design information from the lateral cephalogram to the final design of the appliance. In order to minimize these errors, this study utilizes merged three-dimensional (3D) model images and cephalograms to virtually design custom lingual appliances. In addition to improving the design accuracy, CAD/CAM technology has simplified fabrication by also eliminating soldering process. It provides a mesh type base in the lingual pads to increase bonding strength of the appliance. Additionally, rapid-prototyping technology makes it possible to support undercuts on the lingual pad base, which are not possible with conventional fabrication methods. This study introduces a technique for CAD/CAM fabrication of lingual orthodontic appliances and assesses the final position of the cemented appliance with the planned position on the lateral cephalogram.

## 2. Materials and Methods

This new custom lingual appliance is named kinematics of lingual bar on nonparalleling technique (KILBON). The torque on the maxillary anterior segment is determined by the center of resistance (Cres) and the corresponding retraction force vector. In the sagittal plane, the retraction vector is determined by the vertical position of a palatal temporary skeletal anchorage device (TSAD) and the location of the lever arm [8–10]. When anterior teeth are retracted with palatal TSADs, the lever arm can be located closer to the center of resistance of the maxillary anterior teeth when compared to retraction with buccal TSADs.

**Figure 1:**
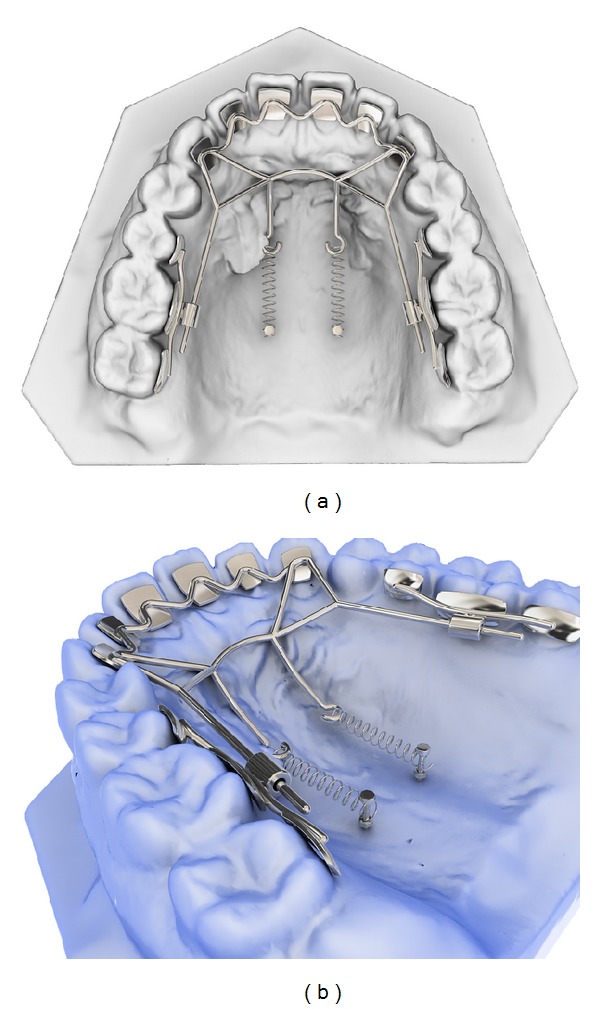
The newly designed lingual retraction system; kinematics of lingual bar on nonparalleling technique (KILBON appliance). The anterior segment is made of lingual pads-wire and lever arms for retraction of the anteriors. Posterior segment has a short tube where guide wire of anterior segment slides through.

The KILBON system consists of the following components: palatal TSADs, anterior lingual pads connected by archwire, and posterior segments (Figure 1). The anterior segment is made of a 0.036-inch wire connected to lingual pads splinting six anterior teeth into a single unit. Two lever arms are attached to the anterior segment and connected to the TSADs with NiTi closed-coil springs for direct retraction. This provides translation of the anterior segment. Each posterior segment is also splinted as one unit, and a short tube is extended from the maxillary first molar. This tube functions as a sliding yoke and vertical hook for intrusion of posteriors. A 0.036-inch guide wire is connected to the retraction hooks and extends distally through the tube. The posterior extension wire gives vertical stabilization to the anterior group of teeth, which prevents unwanted extrusion or intrusion.

**Figure 2:**
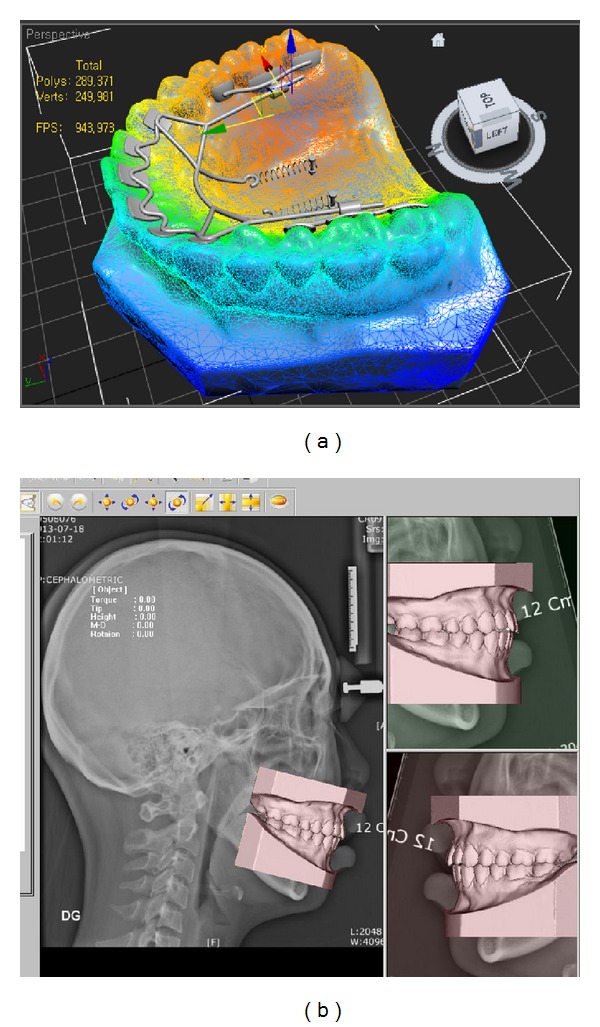
Three-dimensional (3D) model image was obtained by scanning the study model (a) and was registered with cephalograms in the 3Txer software (b).

The KILBON appliance was designed with dental CAD/CAM solution software (3Txer version 2.5, Orapix, Seoul, Korea) and commercial 3D CAD software (Rhinoceros 3D v5.0, Mc Neel & Associates, USA). The 3D image of the study model was produced using a laser scanner (KOD-300 3D, Orapix, Seoul, Korea; accuracy, ±20 *μ*m). The model image was registered with the lateral and frontal cephalograms using the 3Txer software, as described by Choi and colleagues (Figure 2) [11].

**Figure 3:**
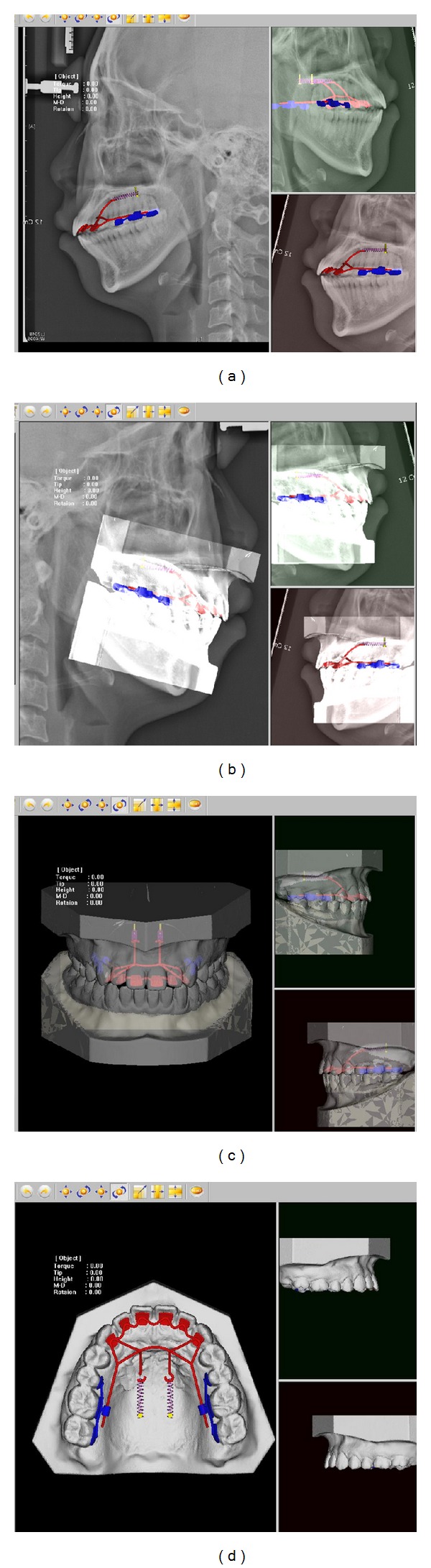
Preliminary design in the 3Txer software. (a) Lever arm length was designed considering center of resistance (Cres) of the 6 anterior teeth on the lateral cephalogram. (b) Registration of 3D model and lateral cephalogram. (c) Appliance design on the frontal view. (d) Occlusal view.

**Figure 4:**
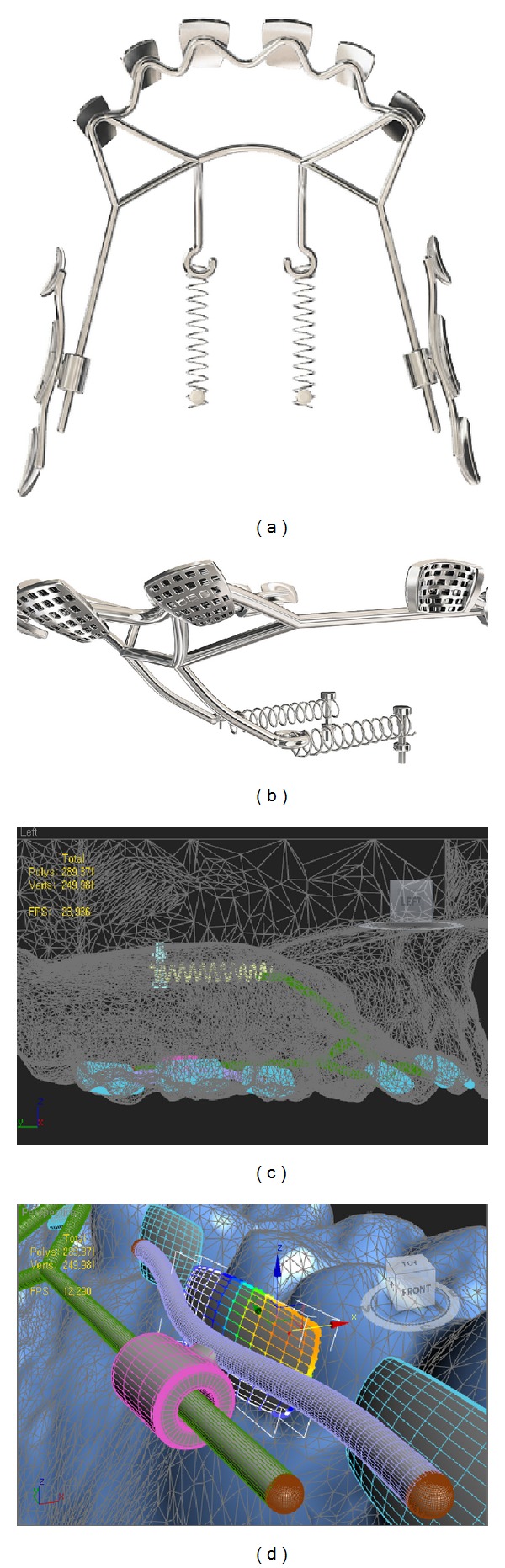
Final design of the appliance in the 3D CAD software. (a) Occlusal view. (b) Retention feature was provided on the lingual pad base. ((c) and (d)) Lingual arch wire was connected to the anterior lingual pads.

On the lateral cephalogram, the center of resistance (Cres) was marked using the measurement function within the software. The placement location of the TSADs and lever arm length were determined based on the desired orientation of the retraction vector. The preliminary construction of the appliance was designed using this information (Figure 3). Design data was exported to commercial 3D CAD software. The bases of the lingual pads were designed according to the lingual anatomy of individual teeth. The exact anatomy of the lingual teeth surfaces was captured using Boolean operator functions within the software. The Boolean operation is a method for obtaining the new shape from two or more existing shapes. The subtraction Boolean operation subtracts one object from another at the point where the objects overlap each other. The resulting object has a surface identical to the lingual surface, and this object is modified to design the lingual pads. To increase bonding strength, repetitive indents were engraved on the pad base. Additional parts of the appliance were designed on the virtual model. The lingual archwire connected to the anterior pads is illustrated in Figure 4.

Before producing a stereolithographic prototype, any defects or voids were examined with reverse engineering software (Rapidform 2006, 3D systems, Seoul, Korea). A prototype of the KILBON appliance was manufactured using a rapid-prototyping machine (Projet MD3000 Plus, 3D systems, Circle Rock Hill, SC, USA). The actual appliance was then manufactured from this stereolithographic prototype using conventional dental casting. The lingual arch component and right and left posterior tube segments were invested using phosphate-bonded investment material and casted with cobalt-chrome alloy. After final finishing and polishing, a transfer jig was fabricated for indirect bonding of the appliance.

**Figure 5:**
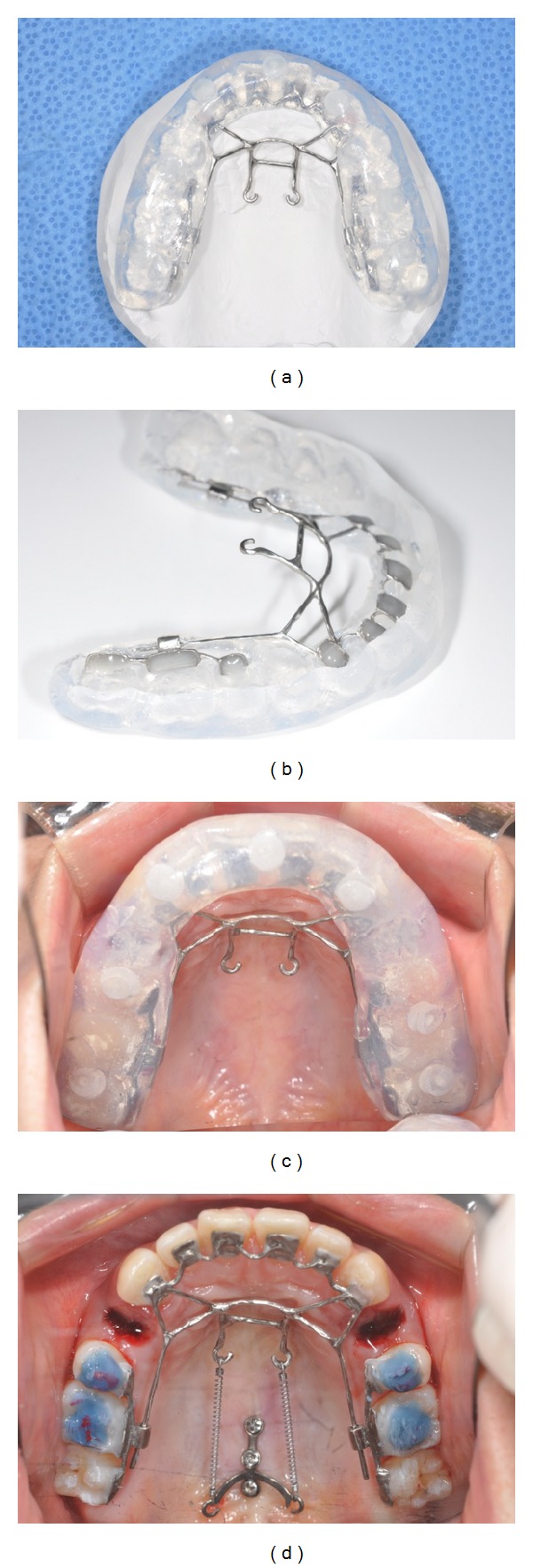
Bonding procedure. (a) Occlusal view of the transfer jig. (b) Resin was applied on the bonding surface of the appliance. (c) The appliance was bonded on the upper arch using a transfer jig. (d) A TSAD was placed and a closed-coil spring was inserted between the TSAD and KILBON appliance.

Prior to trying in the appliance, the tooth surfaces were first etched with 37% phosphoric acid gel (3 M Dental Products, St. Paul, MN, USA) for 30 seconds. In a thin film, a primer (Transbond XT, 3 M, Dental Products, St. Paul, MN, USA) was applied to the etched tooth surface. Then an adhesive paste (Transbond XT, 3 M Dental Products, St. Paul, MN, USA) was applied, and the appliance was positioned using the transfer jig (Figure 5).

**Figure 6:**
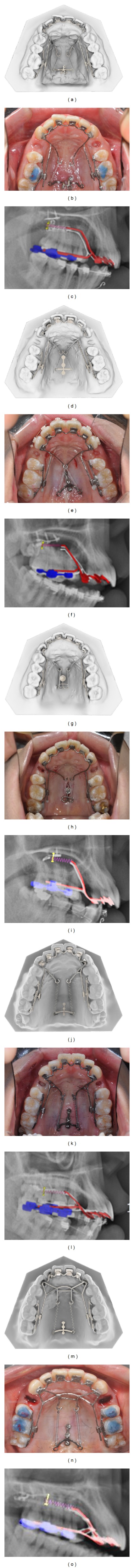
3D KILBON appliance in the CAD software, occlusal photograph and lateral cephalograms. Positional accuracy and rigidity of each design were evaluated by comparing the planned design on the 3D model with a new occlusal photograph and by superimposing a new lateral cephalogram on the initial cephalogram containing design information in the 3Txer software.

To optimize the design with the least distortion during the fabrication process and produce the closest to en-masse anterior retraction, various lever arm designs were applied on five patients. After placing the KILBON appliance, occlusal photographs and lateral cephalograms were taken. Positional accuracy and rigidity of each design were evaluated by comparing the planned design on the 3D model to the new occlusal photograph and through superimposing the new lateral cephalogram on the initial cephalogram containing the design information (Figure 6).

## 3. Results and Discussion

The rigidity and stability of the appliance during retraction varied depending on the lever arm design. When 0.8 mm wire was used for the lever arm (case 1, 17-year-old female), the lever arm bent slightly during en-masse retraction (Figures 6(a) to 6(c)). In cases 2 and 3 (23-year-old females), the wire diameter was increased to 0.9 mm to withstand the retraction force. In these cases, the final position of the appliance deviated slightly from the planned position due to deformation of the anterior lingual wire from postcasting polishing (Figures 6(d) to 6(i)). To overcome this in case 4, an auxiliary wire was added between the extension arm and lever arm to prevent positional change of the lever arm and distortion during casting (22-year-old female, Figures 6(j) to 6(l)). Stability and positional accuracy were improved with this addition. In case 5 (26-year-old female), multiple auxiliary wires were applied to prevent distortion during casting and en-masse retraction, resulting in the best outcome in terms of stability and positional accuracy (Figures 6(m) to 6(o)). In this case, the cemented KILBON appliance maintained the desired position, as planned in the software.

CAD/CAM technology shows a range of promising possibilities in the fabrication of orthodontic appliances. When 3D model and CBCT scans or lateral cephalograms are combined together, the lever arm vector can be virtually designed in the software, and this design information can be saved and exported to the other 3D CAD software. Furthermore, after minor adjustments, this framework design can be used for other patients after minor adjustments. The appliance design can also be converted to fabricate customized brackets following the retraction of the anterior segment. When used with virtual articulation software, premature contacts on the appliance can be eliminated and chair time adjustment is reduced. The treatment result is easily evaluated by comparing registered pre- and posttreatment lateral cephalograms.

Another advantage of the CAD design method is improved bonding of the lingual bracket base. One of the most important factors in the bonding of orthodontic brackets is the type of bracket base [12]. The most commonly used bracket bases are perforated bases, foil mesh bases, photoetched bases, and integrated cast-type bases. The highest resolution of commercially available stereolithographic printers is approximately 0.3 mm [13], which is sufficient for providing the retention feature on the base of a stereolithographic prototype. The base of a metal bonded attachment must be manufactured so that a mechanical interlock between the bonding material and the attachment surface can be achieved [14]. For steel brackets, the bonding material is attached mechanically to the bracket base penetrating into the undercuts provided usually by a fine mesh welded or brazed onto the back of a metal bracket. In another study on CAD/CAM fabricated lingual bracket [15], the smooth surface of the bracket base was sandblasted with aluminum oxide (Rocatec-Pre/Rocatec-Plus, 3 M ESPE, USA) to enhance the retention of the gold alloy bracket. In this study, sandblasting was unnecessary because of the built-in retention features designed in the bracket base. 3D scanning of the models with a high-resolution scanner enabled individualization of the brackets using a precise image of the lingual surface. This is necessary since the lingual surfaces of teeth vary much more widely than labial surfaces [16–18]. This method also minimizes bracket thickness [19].

The vertical height of retraction hooks controls the resulting movement of the anterior teeth, resulting in tipping, bodily movement, or lingual root movement during retraction. The double J retractor introduced two lever arm hooks for space closure [20]. The anterior long lever arm hooks were designed to pass the line of action of this force through the center of resistance. Unlike traditional lingual brackets and archwire, the one-body structure of the lingual pads and lingual wire eliminated any wire play in the brackets and prevented loss of torque control during retraction. Furthermore, the single-body design reduced the high cost of lab fees for lingual brackets.

The KILBON appliance was fabricated by casting a stereolithographic prototype. During casting, the fragile parts of the appliance are subject to distortion and require reinforcement. In most cases, conventional dental casting utilizes a wax pattern, and distortion of this casting can be attributed to distortion of the wax pattern. The stereolithographic prototype is much more rigid, and therefore distortion is reduced in comparison to the traditional lost wax technique. However, some distortion can be caused by hardening of the investment around the prototype, whereby setting and hygroscopic expansion could lead to uneven deformation of the walls of the prototype. This depends on the thickness and configuration of the prototype. The addition of auxiliary wire and selection of the appropriate wire diameter result in less distortion of the appliance.

In this study, the KILBON appliance was applied on five patients. A greater sample size is required for a more thorough evaluation. Further studies are required to optimize the angulation of the lever arm and resulting retraction vectors of the anterior and posterior segments.

## 4. Conclusions

CAD technology, equipped with merged image of 3D model image and cephalograms or CBCT scans, enables improved accuracy of orthodontic appliance design. Using computer-assisted design and manufacturing of the KILBON appliance, the following results were obtained:

1. the use of auxiliary wires reduced the distortion of the appliance during casting;
2. wire diameter should be larger than 0.9 mm to withstand retraction force.
